# High-Intensity Interval Training Ameliorates Tramadol-Induced Nephrotoxicity and Oxidative Stress in Experimental Rats

**DOI:** 10.7759/cureus.62518

**Published:** 2024-06-17

**Authors:** Najmeh Sadat Hosseini, Sara Shirazpour, Mahla Zangiabadizadeh, Hamideh Bashiri, Shahriar Dabiri, Gholamreza Sepehri, Manzumeh Shamsi Meymandi

**Affiliations:** 1 Neuroscience Research Center, Institute of Neuropharmacology, Kerman University of Medical Sciences, Kerman, IRN; 2 Physiology Research Center, Institute of Basic and Clinical Physiology Sciences, Kerman University of Medical Sciences, Kerman, IRN; 3 Pathology and Stem Cell Research Center, Kerman University of Medical Sciences, Kerman, IRN

**Keywords:** high-intensity interval training (hiit), inflammation, kidney function, apoptosis, oxidative stress, tramadol

## Abstract

Introduction: Tramadol (TRA) is an opioid analgesic widely prescribed for moderate-to-severe pain; however, its abuse and chronic use have been associated with kidney damage. Considering the protective role of exercise training in reducing organ damage, this study aimed to assess the influence of high-intensity interval training (HIIT) on a male rat's kidney following chronic TRA administration.

Methods: In this experimental study, 30 male Wistar rats were assigned to the following groups: control (CON; animals received normal saline five days a week in the first month and three days a week in the second month), exercise (EXE; animals conducted HIIT training according to exercise protocol five days a week for two months), TRA (animals received TRA 50 mg/kg (i.p.) as described for the CON group), EXE-TRA (animals received TRA and conducted exercise protocol), and EXE-SL (animals received normal saline and conducted exercise protocol). Then, serum IL-6 and IL-10 levels, tissue malondialdehyde (MDA), total antioxidant capacity (TAC), glutathione peroxidase (GPx), superoxide dismutase (SOD), and levels of albumin, urea, and creatinine (CR), along with pathological changes in the kidney, were measured. A p-value of <0.05 was considered significant using GraphPad Prism v.9 (GraphPad Software, La Jolla, California, USA).

Results: The inflammatory cytokines IL-6 and IL-10 were significantly increased in the EXE and EXE-TRA groups compared to the TRA group. Chronic administration of TRA in the TRA group decreased antioxidant indicators TAC, GPx, and SOD in kidney tissue while increasing oxidative stress MDA compared to the CON group (p<0.05). In contrast, the EXE-TRA group showed higher levels of TAC, GPx, and SOD, while MDA decreased compared to the TRA group. Additionally, serum levels of urea and CR were increased in the TRA group compared to the CON group, whereas these levels were decreased in the EXE-TRA group compared to the TRA group. The inflammatory effect of HIIT training, due to severe hyperemia and mild inflammatory cell infiltration, was seen in all EXE groups. Pathological findings confirmed TRA-induced kidney damage through moderate hyaline cast presence and severe apoptosis in the TRA group. Other findings were in line with the above results.

Conclusion: These findings confirm the nephrotoxicity of chronic use of TRA through biochemical and oxidative markers and pathological outcomes. In addition, the result suggests that HIIT has the potential to mitigate the detrimental effects of TRA through reversing biochemical and oxidative markers, including TRA-induced apoptosis. Consequently, considering its restorative properties, HIIT could be explored as a prospective nephroprotective approach for long-term TRA treatment.

## Introduction

Escalating reports concerning a substantial increase in tramadol (TRA) misuse have been well-documented worldwide. TRA is a centrally acting analgesic with opioid-agonist properties used to treat moderate-to-severe acute and chronic pain [1]. Long-term TRA administration for pain treatment and its abuse as an alternative for drug seekers have made TRA one of the most commonly prescribed opioids worldwide [2].

TRA is rapidly absorbed after oral administration, and its distribution half-life is approximately 1.7 hours. Its high total distribution volume of 306 L indicates a strong tissue affinity. In the liver, TRA undergoes extensive metabolism through O- and N-demethylation and conjugation reactions, producing glucuronides and sulfates. The kidneys excrete metabolites resulting from this biotransformation [3].

The availability of TRA and its low cost have led to its widespread abuse in many countries [4]. The statistics indicate that more people aged 12 or older use TRA for non-medical purposes in their lifetime [4]. The high consumption of TRA causes the accumulation of active metabolites in the body, which results in a reduced rate of clearance, leading to toxicity [5]. Several experimental studies have indicated that reactive oxygen species (ROS) and inflammation production are the main mechanisms of TRA-induced toxicity [6]. Indeed, toxicity induced by TRA results in the activation of the inflammatory reaction and a significant increase in the production of proinflammatory cytokines [7]. TRA could increase the levels of malondialdehyde (MDA), nitric oxide, and monoamine neurotransmitters in the brain [8]. Moreover, it can reduce activity and antioxidant defense and antiapoptotic agent expression and increase apoptotic and inflammation gene expression [9]. Furthermore, studies demonstrated that the administration of TRA increased serum levels of urea, creatinine (CR), uric acid, and nuclear factor kappa-light-chain enhancer of activated B cells (NF-κB) [6].

There is evidence that TRA can be toxic to the kidneys when consumed in high doses or over a long period of time [10]. Several studies have shown that TRA induces renal injury by increasing the aggregation of inflammatory cells and oxidative stress [10-12]. Therefore, it appears that the reduction of apoptosis, oxidative stress, and inflammation is the most effective way to decrease the progression of kidney damage following TRA administration. In this regard, exercise (EXE) training has emerged as a promising means of reducing apoptosis, ROS, and inflammation [13,14]. High-intensity interval training (HIIT) has been demonstrated to effectively reduce oxidative stress and inflammation in the kidney while enhancing antioxidant capacity [14]. The high-intensity intervals followed by recovery periods in HIIT stimulate the production of antioxidants, enhancing the body's ability to combat oxidative damage and promoting a more balanced immune response. Compared to other regular EXEs, HIIT offers shorter and more time-efficient workouts, yet it provides similar or even greater benefits in reducing oxidative stress and inflammation and improving antioxidant levels [14]. Mechanisms responsible for the beneficial effects of HIIT in the kidney include training-induced upregulation of superoxide dismutase (SOD), catalase, and reduced ROS in the kidney [14]. Other studies have shown that regular EXE positively reduces most of the reported inflammatory markers, including CRP and IL-6 [13-15]. Also, it has been found that EXE training raises IL-10 levels, indicating a reduction in systemic inflammation [14,15]. Moreover, recent studies have shown that EXE training significantly decreases the protein levels of proapoptotic signaling and increases the protein level of antiapoptotic proteins [16]. Previous studies have extensively explored the association of kidney function indicators with both TRA administration and HIIT [10,12,15]. Additionally, studies have indicated that chronic use of TRA can lead to significant renal impairment, as evidenced by elevated levels of serum urea and CR, which are critical indicators of kidney function [10,12]. Furthermore, HIIT is associated with lower serum urea and CR levels, reflecting improved kidney function [15]. These findings suggest that while TRA poses risks to renal health, HIIT offers a protective effect, highlighting its potential as a therapeutic intervention for maintaining kidney function.

Several studies have shown that EXE reduces inflammatory and oxidative indices, and TRA chronic use causes toxicity in various organs, including the kidney. To the best of our knowledge, there are no studies that investigated the effect of training in reducing kidney damage following TRA consumption. (The preprint of this article was previously posted on Research Square [17].) This study was designed to evaluate the impacts of HIIT on the male rat's kidney functions following chronic use of TRA administration.

## Materials and methods

Animal care and drugs

**Figure 1:**
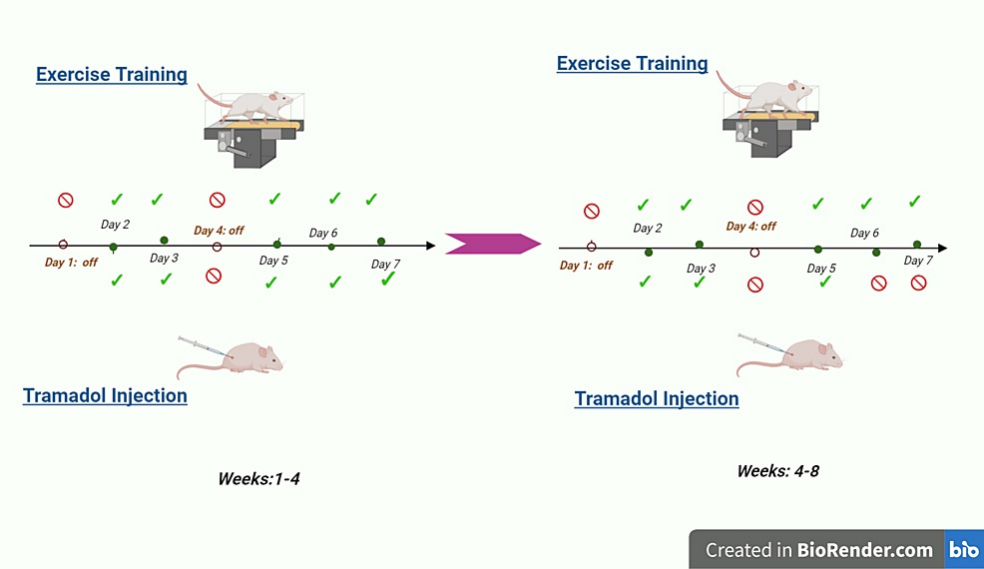
Procedure protocol Image Credit: Author, created in BioRender.com

**Figure 2:**
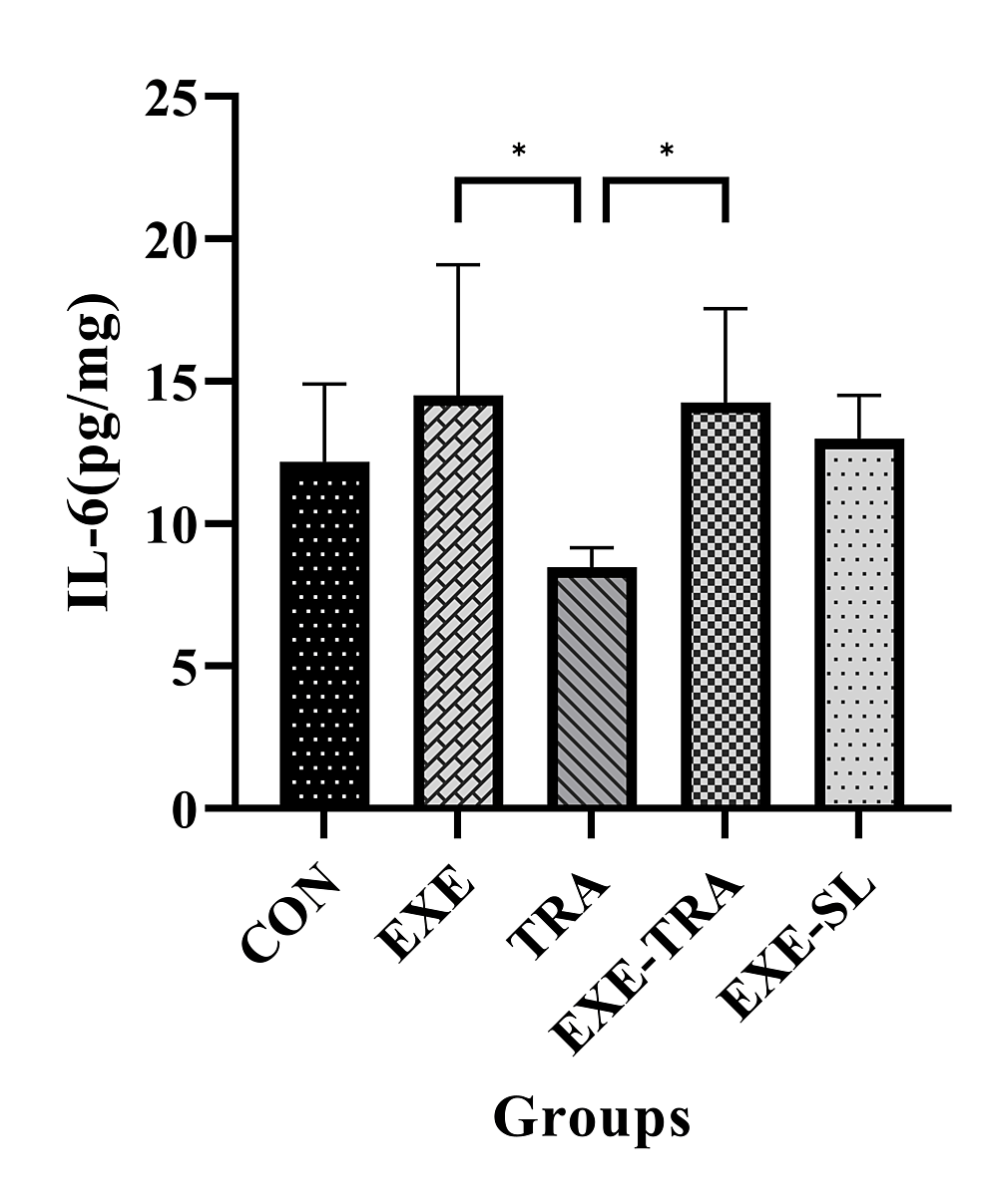
Effect of eight weeks of HIIT on IL-6 in tissue Data are presented as mean ± SD for n=5 in each group. In the group of animals receiving TRA administration, the IL-6 level decreased in comparison to both EXE alone and the EXE-TRA group (p<0.05). * p<0.05, CON: control, EXE: exercise, TRA: tramadol, EXE-TRA: exercise-tramadol, EXE-SL: exercise-normal saline

The Animal Research Ethics Committee of Kerman University of Medical Sciences granted approval for this project (approval number: IR.KMU.AH-AEC.1402.014) in accordance with the Declaration of Helsinki of animal rights and the National Institutes of Health Guide for Care and Use of Laboratory Animals (publication number: 85-23, revised 1985). TRA (Kamud Drugs, India) and 30 male Wistar rats (weight: 150-200 g) were used in the experiments. The rats were maintained in a 12-hour light, 12-hour dark cycle at a temperature of 22°C with free access to water and food. All animals were randomly divided into five groups (n=5): (1) control group (CON; animals received normal saline via intraperitoneal (i.p.) injection (10 mL/kg) five days a week for the first month and three days a week for the second month; Figure 1), (2) EXE group (animals performed HIIT five days a week for eight weeks), (3) TRA group (animals received TRA (50 mg/kg, i.p.) following the same schedule as the CON group [18]), (4) EXE-TRA group (animals received TRA (50 mg/kg, i.p.) and performed HIIT as described for the EXE group), and (5) EXE-normal saline group (EXE-SL; animals performed the HIIT protocol and received normal saline as described for the CON group).

All i.p. injections were administered at a volume of 10 mL/kg body mass. Additionally, all experiments and statistical analyses were performed by a researcher who was blinded to the study conditions.

EXE group protocol

Before the experiments, all animals were familiarized with the motorized treadmill (made by Tajhiz Gostare Omid Iranian, Iran). The rats ran on the treadmill at 8 m/min with an incline of 0%, 10 to 15 min/day, five times a week for two weeks. The EXE, EXE-TRA, and EXE-SL groups then underwent an incremental running assessment to establish their maximum running speed (VMax). This involved running at a speed of 6 m/min for two minutes, with an increase of 2 m/min every two minutes until exhaustion. The last tolerated speed was recorded as VMax. The training protocol was then carried out five times a week for eight weeks, with VMax measured weekly and used to calculate relative speed for the next two weeks. A three-minute warm-up and cool-down were performed before and after each EXE session, respectively, with no incline used throughout. The CON remained inactive on the treadmill belt for the same duration (Table 1) [19].

**Table 1:** EXE protocol The slope was 0 and the frequency was 5 in all weeks EXE: exercise

Week	Intervals	High-intensity interval duration (min)	Low-intensity interval duration (min)	High-intensity interval velocity (%VMax)	Low-intensity interval velocity (%VMax)	Total EXE time in a session (min)
1	4	2	1	80	50	12
2	4	2	1	85	50	12
3	6	2	1	85	50	18
4	6	2	1	90	50	21
5	8	2	1	90	50	24
6	8	2	1	95	50	24
7	10	2	1	95	50	30
8	10	2	1	100	50	30

Sample collection

The animal was euthanized using a lethal dose of xylazine (10 mg/kg) and ketamine (75 mg/kg) via intraperitoneal injection 48 hours after the last EXE session to collect blood and tissue samples. A blood sample was withdrawn by heart puncture, and serum separation was performed by centrifugation (Novin Tashkhis, Co., Iran) at 5000×g for 15 minutes at 4°C. Kidney tissue was extracted and immediately frozen using liquid nitrogen for molecular analysis at -80°C.

Tissue homogenization

After removal, the kidneys were rapidly washed in 50 ml of ice-cold PBS, cut into small pieces, and placed into ice-cold homogenization buffer (250 mM sucrose, 1 mM EDTA, 10 mM NaOH-Hepes, pH 7.4) containing protease inhibitors (Roche cOmplete protease inhibitor cocktail). Minced kidneys were homogenized in a Dounce homogenizer for 10 passes (Navand Salamat, Co., Iran). The homogenate was then centrifuged at 1000 ×g for 10 minutes, and the supernatant was centrifuged at 200,000 ×g again for one hour at 4°C for the fraction process of membrane compartments. The pellet was resuspended in a homogenization buffer.

Measurement of inflammatory indicators

The cytokines, IL-6 and IL-10, were measured in tissue by ELISA kits (Karmania Pars Gene Company, Mashhad, Iran) according to the manufacturer’s recommended procedure. The procedure was carried out in accordance with the instructions provided by the manufacturer. Initially, 50 µL of both samples and standards were introduced into precoated plates and left to incubate for 16-20 hours at a temperature of 4°C. This was followed by three washing cycles. Subsequently, 50 µL of conjugated antibody was added to the plates and allowed to incubate at room temperature for one hour, followed by another four washing cycles. Then, HRP avidin (50 µL) was added to the plates and left to incubate in a shaker incubator at room temperature for 30 minutes while being agitated at a speed of 200 rpm. After four more washing cycles, 90 µL of substrate was added and left to incubate at room temperature for 15 minutes. The reaction was terminated, and the optical density was measured at a wavelength of 450 nm. All factors were measured using a rotator device (Novin Tashkhis, Iran), the EMP M-201 ELISA reader (Chengdu Empsun Medical Technology Co., Ltd., China), and the ELISA microplate washer (Shenzhen Emperor Electronics Co., Shenzhen, China).

Measurement of oxidative stress indices

After preparing kidney tissue samples, oxidative stress indices were measured. MDA, an indicator of lipid peroxidation, was measured using the thiobarbituric acid (TBA) assay. When MDA reacted with TBA in the presence of a trichloroacetic acid-TBA-hydrochloric acid reagent, it produced a pink color. The absorbance of this pink complex was then measured at 535 nm. The total antioxidant capacity (TAC) of serum was determined using the ferric reducing antioxidant power (FRAP) assay. In this assay, at low pH, plasma reduces the ferric tripyridyltriazine (Fe III-TPTZ) complex to its blue-colored ferrous (Fe II) form, which has a maximum absorbance of 593 nm. The intensity of the blue color is proportional to the antioxidant capacity of the sample. Specifically, 5 μL of plasma was mixed with 70 μL of FRAP reagent, incubated at 37°C for five minutes, and the absorbance was read at 593 nm using distilled water as a blank. All the tests were performed using the Epoch microplate reader (BioTek Instruments, VT, USA). The activity of SOD was measured using the Randox kit (Randox Laboratories Ltd., UK; Cat No. RS504) following the manufacturer's protocol. SOD catalyzes the dismutation of superoxide radicals (O2-) into hydrogen peroxide and oxygen. In the Randox assay, superoxide ions generated by xanthine oxidase convert nitroblue tetrazolium (NBT) to NBT-diformazan, which absorbs light at 560 nm. SOD decreases the formation rate of NBT-diformazan by reducing the concentration of superoxide ions. The activity of SOD was quantified by measuring the reduction in the formation of NBT-diformazan in the sample. The activity of glutathione peroxidase (GPx) was assessed using the Randox kit (Randox Laboratories Ltd., UK; Cat No. SD125). This assay measures GPx activity indirectly through a coupled reaction with glutathione reductase, which regenerates reduced glutathione from its oxidized form generated by GPx. The decrease in absorbance at 340 nm (A340) due to the oxidation of NADPH to NADP+ was used to determine the activity of GPx.

Measurement of kidney function indicators

Serum urea was measured by the urease Berthelot colorimetric method, serum CR was quantified using the alkaline picrate colorimetric method, and serum albumin (Alb) was measured by the ELISA method. We used the Epoch microplate reader.

Histopathology

The kidney of a rat was collected and fixed with 10% formaldehyde embedded in tissue paraffin. The resulting kidney tissue sections were then stained using the standard H&E method. These H&E-stained slides were examined using a bright field microscope (Olympus BX53, Japan) and then photographed (Olympus DP73, Japan).

H&E staining protocol

The kidney was fixed and embedded in paraffin. Kidney sections were cut at a distance of 4-5 μm from the lung tissues embedded in paraffin and mounted on glass slides. After removing paraffin and rehydrating, tissue sections measuring 4 µm were washed in distilled water and then treated with a series of solutions, including 1% potassium permanganate (Merck, Darmstadt, Germany) for two minutes, followed by 2.5% oxalic acid (Merck, Darmstadt, Germany) for one minute, 2% iron alum (Merck, Darmstadt, Germany) for one minute, Gomori’s solution for three minutes, 10% formalin (Merck, Darmstadt, Germany) for two minutes, gold chloride (1:500; Merck, Darmstadt, Germany) for three minutes, 3% potassium metabisulfite (Merck, Darmstadt, Germany) for one minute, and finally, 3% sodium thiosulfate (Merck, Darmstadt, Germany) for another minute. Each slide section was rinsed with distilled water before immersion in the solution. Tissue sections were then examined under a light microscope and photographed as JPG files.

After H&E staining, inflammation, apoptosis, hyperemia, degeneration, and tissue changes were evaluated by two blinded pathologists. Then the mean score of kidney damage from four adjacent incisions was obtained. The severity of kidney damage was determined using a 5-point scoring system. Scoring standards are 0 - no damage, 1 - minimal (0-5% involvement), 2 - mild (5-25% involvement), 3 - moderate (25-75% involvement), and 4 - severe (75-100% involvement).

Statistical analysis

GraphPad Prism v.9 (GraphPad Software, CA, USA) was used for statistical analysis. The Shapiro test determined the normal distribution of the data. Firstly, to estimate the interaction of EXE and TRA, a two-way ANOVA was performed. Then a one-way ANOVA and a Tukey post hoc test were used to investigate the significant differences in the research groups. Data were presented as mean ± SD, and the significance level was considered less than 0.05.

## Results

Effect of HIIT on IL-6 and IL-10 in kidney tissue following eight weeks of TRA administration

EXE (but not TRA) showed a significant effect on IL-6 level (F(1.21)=9.25, p=0.006). No differences were found between the CON group and EXE and/or TRA groups. However, in the TRA group, the level of IL-6 decreased compared to the EXE and EXE-TRA groups (p<0.05). Although EXE did not change the level of IL-6, it repaired the IL-6 reduction induced by TRA, confirming the main effect of EXE on IL-6 (Figure 2).

**Figure 3:**
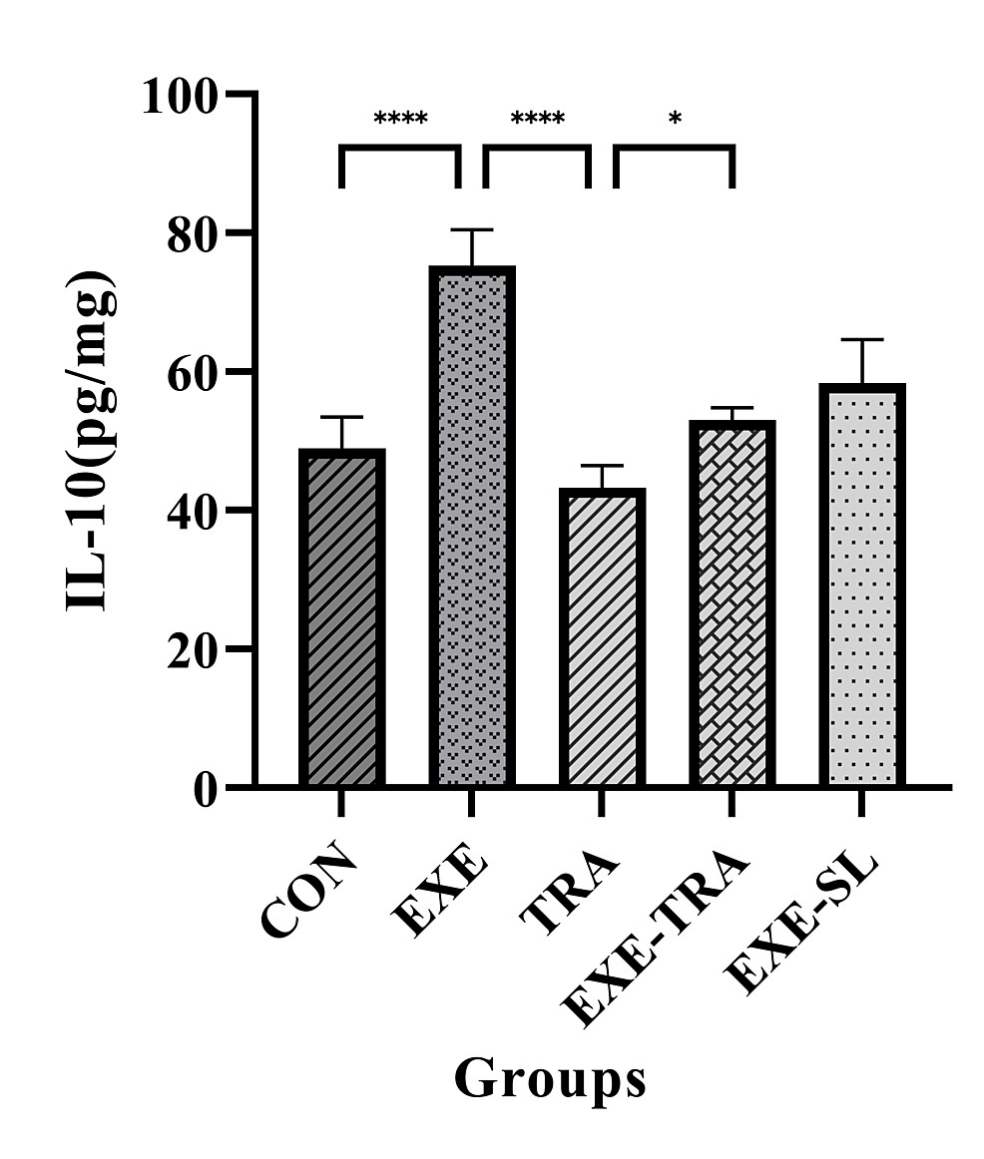
Effect of EXE on IL-10 in tissue Data are presented as mean ± SD for n=5 in each group. The level of IL-10 increased in the EXE group compared to the CON and TRA groups (p<0.0001). We also observed a significant difference between the EXE-TRA and TRA groups (p<0.05). * p<0.05, **** p<0.0001. CON: control, EXE: exercise, TRA: tramadol, EXE-TRA: exercise-tramadol, EXE-SL: exercise-normal saline

Both EXE and TRA showed significant effects on IL-10 (F (1.21)=10.1, p=0.004 and F(1,21)=20.6, p=0.004, respectively). The level of IL-10 in the EXE group increased compared to the CON and TRA groups (p<0.0001). TRA alone was not able to change tissue level of IL-10 significantly. However, interestingly, EXE increased the level of IL-10 even with TRA since a significant difference was also found between the EXE-TRA and TRA groups (p<0.5; Figure 3).

Effect of HIIT on MDA, TAC, GPX, and SOD in kidney tissue following eight weeks of TRA administration

**Figure 4:**
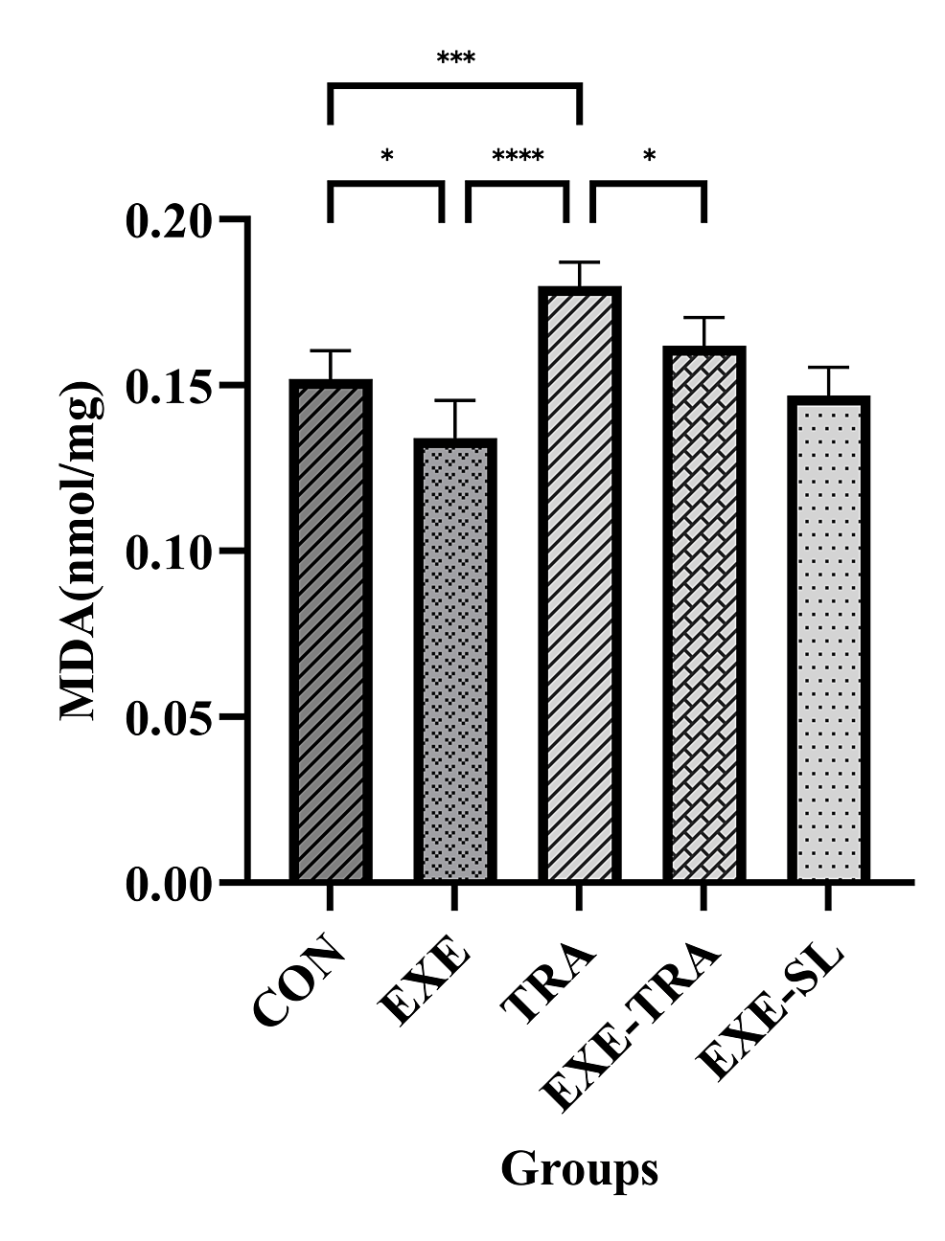
Effect of eight weeks of HIIT on MDA in tissue Data are presented as mean ± SD for n=5 in each group. The MDA level was significantly lower in the EXE-TRA group compared to the TRA group (p<0.05). However, it was higher in the EXE-TRA group compared to the EXE group, indicating a partial attenuation of MDA levels with the EXE but still higher than the baseline level observed with EXE alone. * p<0.05, *** p<0.001, **** p<0.0001, CON: control, EXE: exercise, TRA: tramadol, EXE-TRA: exercise-tramadol, EXE-SL: exercise-normal saline

The two-way ANOVA showed a significant effect of TRA (F(1.21)=14.3, p=0.001) and EXE (F(1.21)=34.6, p=0.000) on MDA levels. The MDA level in kidney tissue significantly increased in the TRA group (p<0.001) and decreased in the EXE group (p<0.05) compared to the CON group. Additionally, the MDA level was decreased in the EXE-TRA group compared to the TRA group (p<0.05) and increased compared to the EXE group (p<0.001) (Figure 4). These results demonstrate that HIIT is able to prevent the increase in MDA caused by TRA.

TRA and EXE had a significant effect on SOD levels (F(1.21)=7.8, p=0.011 and F(1.21)=19.9, p=0.000, respectively). However, no interaction between them was observed. Similarly, for TAC levels, both TRA and EXE had a significant effect (F(1.21)=12.6, p=0.002 and F(1.21)=28.6, p=0.000, respectively). Additionally, a significant interaction of EXE and TRA was observed on TAC levels (F(1.21)=4.8, p=0.04). The same result was observed for GPx, where the effect of TRA (F(1.21)=15.7, p=0.001) and EXE (F(1.21)=28.3, p=0.000) were both significant.

**Figure 5:**
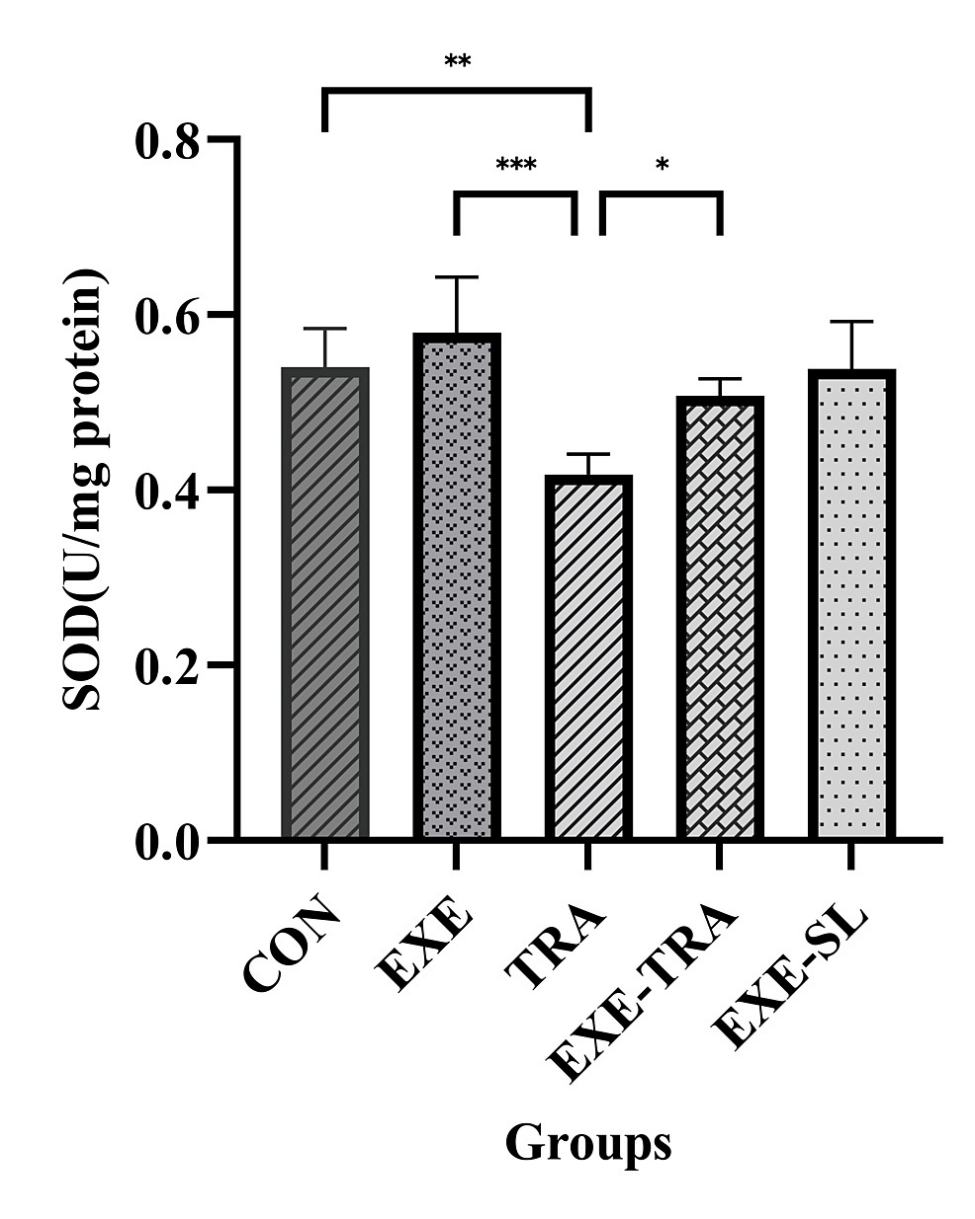
Effect of eight weeks of HIIT on SOD in tissue Data are presented as mean ± SD for n=5 in each group. Both TRA and EXE had significant effects on SOD. TRA resulted in a significant decrease in SOD compared to the EXE, CON, and EXE-TRA groups, while EXE led to a significant increase in SOD. * p<0.05, ** p<0.01, *** p<0.001, CON: control, EXE: exercise, TRA: tramadol, EXE-TRA: exercise-tramadol, EXE-SL: exercise-normal saline

**Figure 6:**
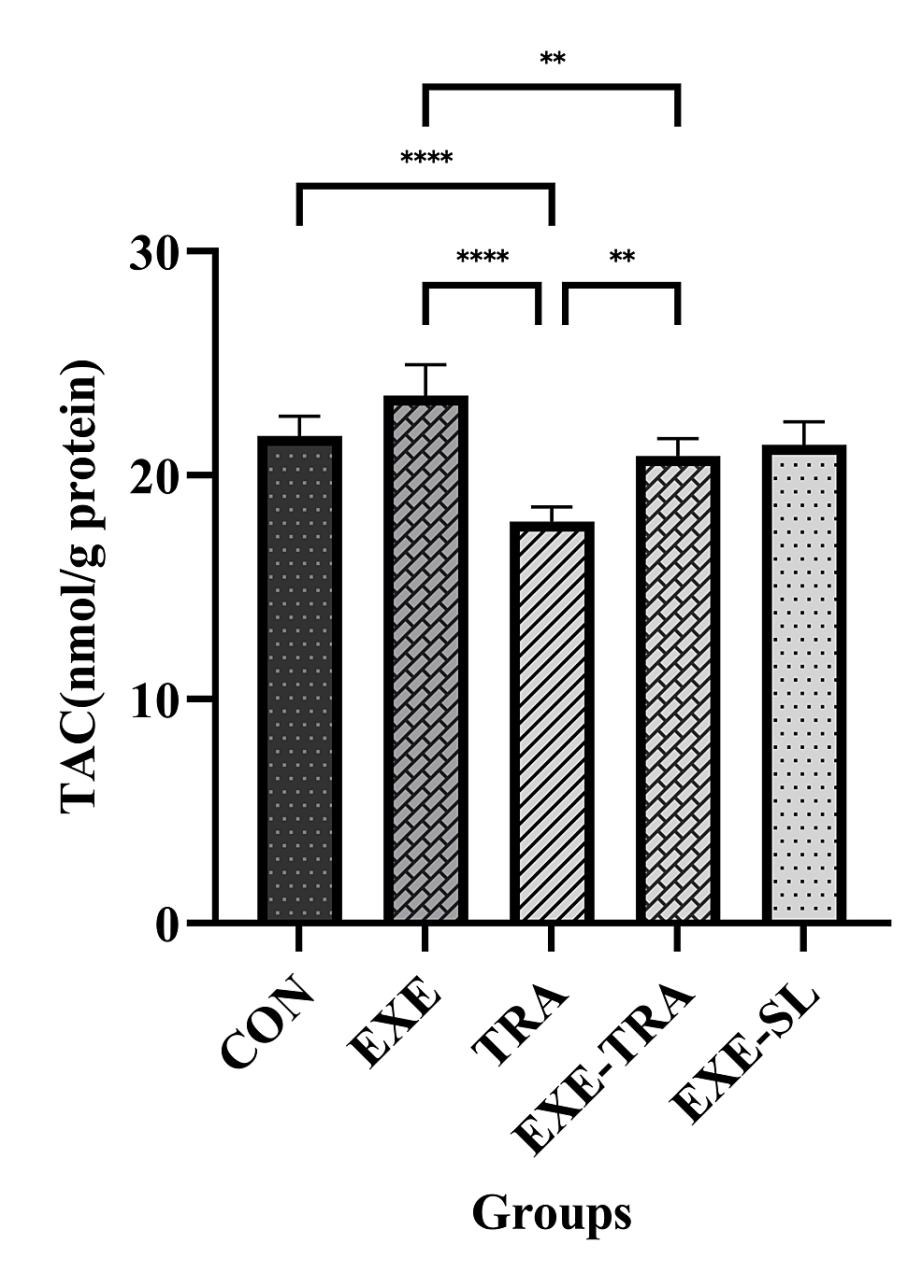
Effect of eight weeks of HIIT on TAC in tissue Data are presented as mean ± SD for n=5 in each group. Both TRA and EXE significantly impacted TAC levels. TRA caused a notable reduction in TAC when compared to the EXE and CON groups, whereas EXE significantly boosted TAC levels in the EXE-TRA group compared to the TRA group. ** p<0.01, **** p<0.0001, CON: control, EXE: exercise, TRA: tramadol, EXE-TRA: exercise-tramadol, EXE-SL: exercise-normal saline

**Figure 7:**
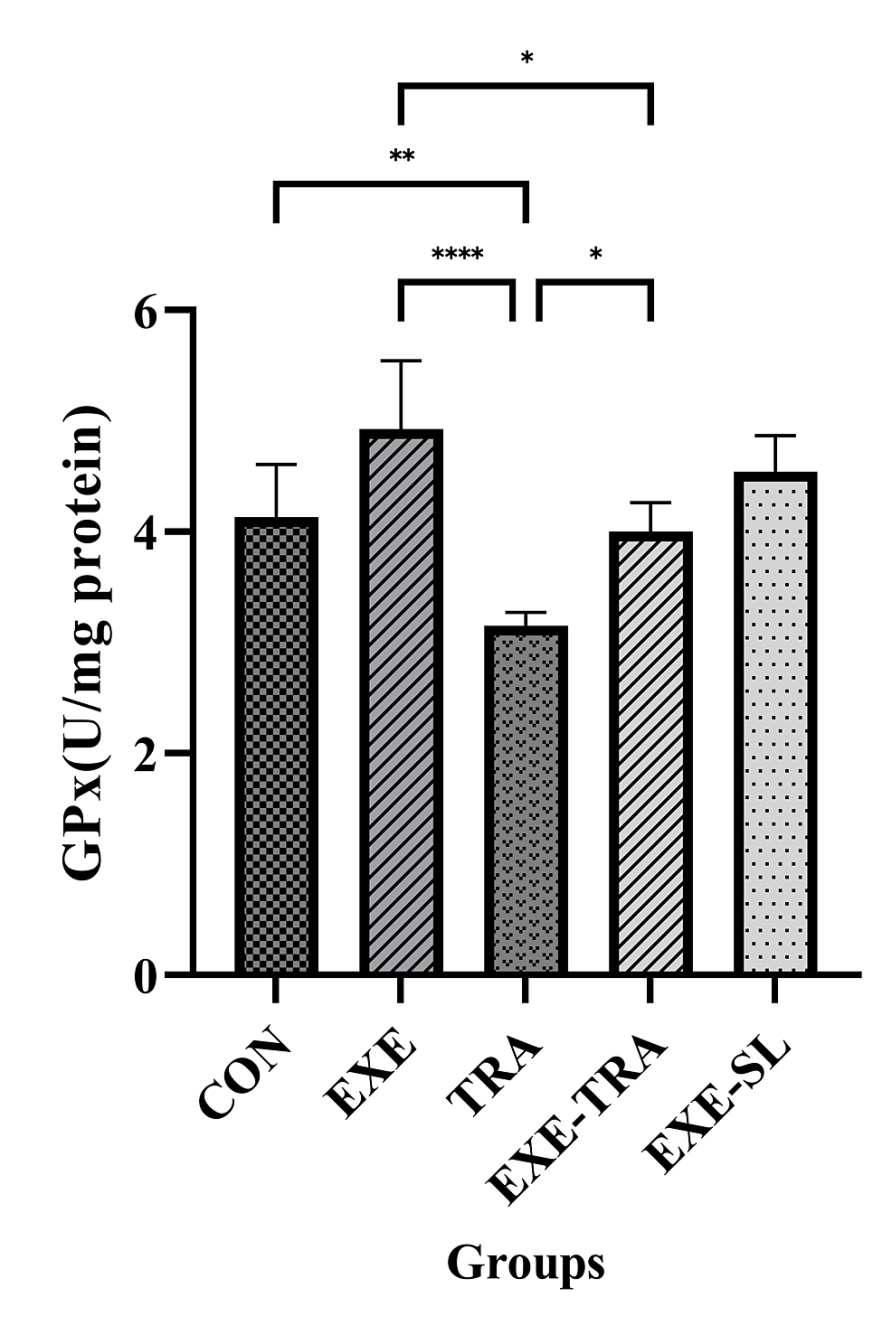
Effect of eight weeks of HIIT on GPX in tissue Data are presented as mean ± SD for n=5 in each group. TRA caused a notable reduction in GPx levels compared to the EXE and CON groups. In contrast, EXE significantly increased GPx levels in the EXE-TRA group compared to the TRA group. * p<0.05, ** p<0.01, **** p<0.0001, CON: control, EXE: exercise, TRA: tramadol, EXE-TRA: exercise-tramadol, EXE-SL: exercise-normal saline

Regarding these kidney oxidative stress variables, the results showed that SOD, TAC, and GPx levels in the TRA group decreased significantly compared to the CON group (p<0.01, p<0.001, and p<0.01, respectively) and the EXE group (all p<0.001) (Figures 5-7). In the EXE-TRA group, EXE significantly increased SOD, TAC, and GPx levels compared to the TRA group (all p<0.05). However, TRA decreased the level of TAC (p<0.01) and GPx (p<0.05) in the EXE-TRA group compared to the EXE group (Figures 6-7). It appears that EXE can reverse the effect of TRA on SOD, TAC, and GPx levels following TRA administration to the CON group level.

Effect of HIIT on Alb, Urea, and CR in serum following eight weeks of TRA administration

**Figure 8:**
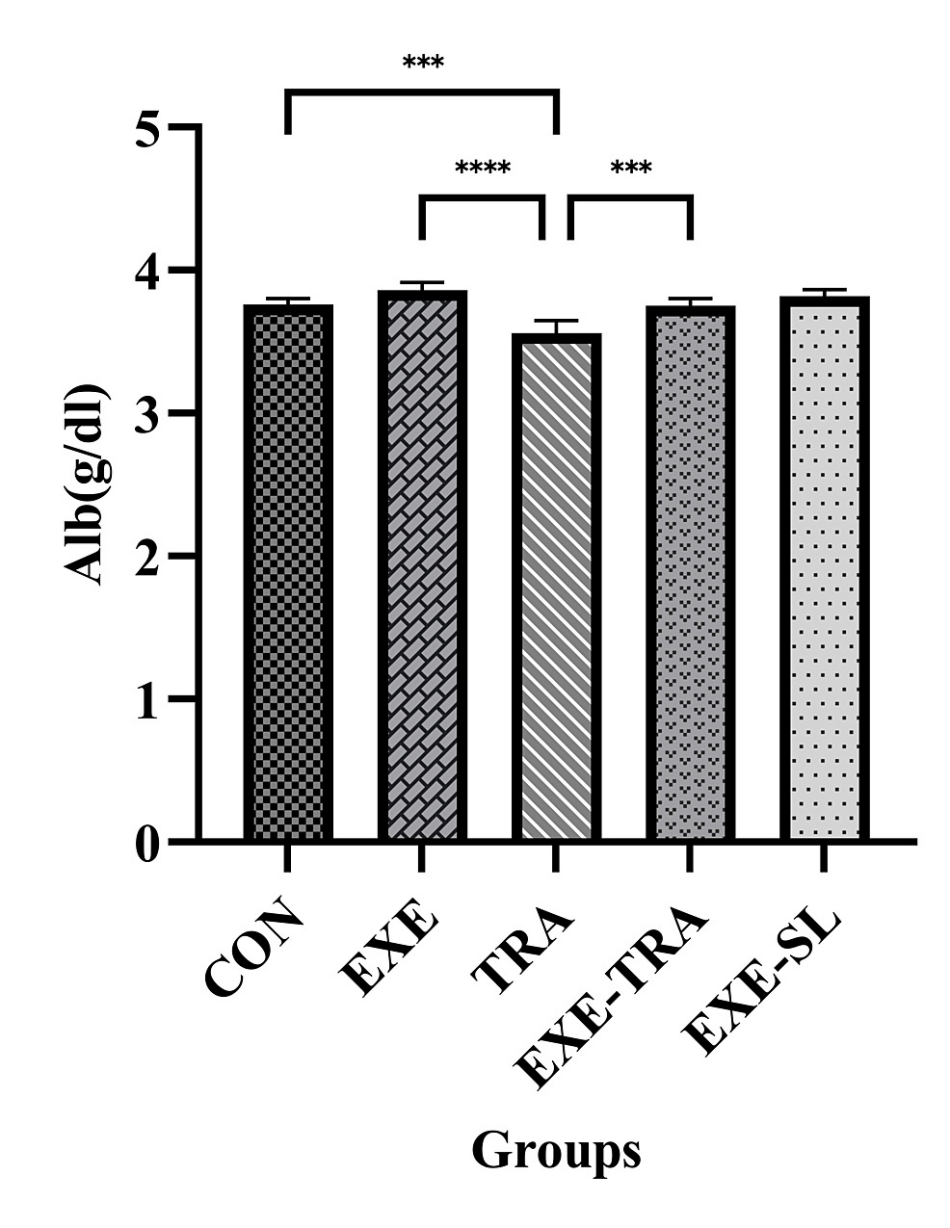
Effect of eight weeks of HIIT on Alb in serum Data are presented as mean ± SD for n=5 in each group. Our results show that Alb levels were significantly lower in the TRA group compared to the CON and EXE groups. Additionally, EXE raised Alb levels in the EXE-TRA group compared to the TRA group. *** p<0.001, **** p<0.0001, CON: control, EXE: exercise, TRA: tramadol, EXE-TRA: exercise-tramadol, EXE-SL: exercise-normal saline

Consistent with our results, EXE and TRA had a significant effect (F(1.21)=25.4, p=0.000) and (F(1.21)=38.9, p=0.000) on Alb levels. Additionally, a significant interaction was observed between them (F(1.21)=6.9, p=0.016). Alb was significantly decreased in the TRA group compared to the CON (p<0.001) and EXE (p<0.0001) groups. Moreover, EXE increased Alb levels in the EXE-TRA group compared to the TRA group (p<0.001), while no difference was observed between the EXE-TRA and CON groups, confirming the beneficial effect of EXE even in the presence of TRA (Figure 8).

The level of urea was affected by both EXE and TRA (F(1.21)=412.9, p=0.000 and F(1.21)=537.7, p=0.000, respectively). The interaction between them was also significant (F(1.21)=288.1, p=0.000). Similar results were found for CR levels, where the effect of EXE (F(1.21)=11.8, p=0.002) and TRA (F(1.21)=9.0, p=0.007) was significant. The interaction between them was also significant (F(1.21)=11.8, p=0.002).

**Figure 9:**
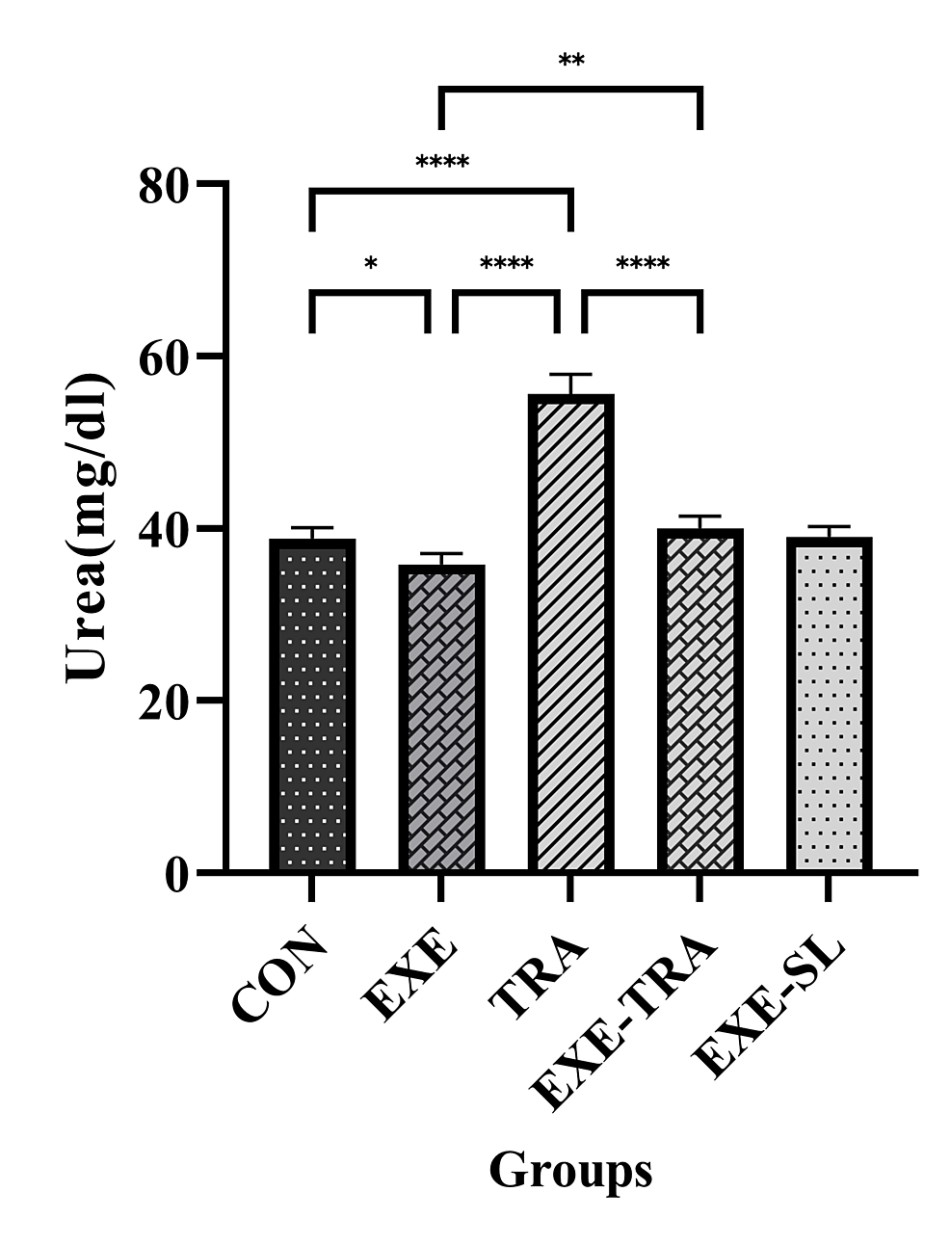
Effect of eight weeks of HIIT on urea in serum Data are presented as mean ± SD for n=5 in each group. Our results indicate a significant increase in urea levels in the TRA group compared to all other groups. Furthermore, EXE decreased urea levels in the EXE-TRA group compared to the TRA group. * p<0.05, ** p<0.01, **** p<0.0001, CON: control, EXE: exercise, TRA: tramadol, EXE-TRA: exercise-tramadol, EXE-SL: exercise-normal saline

**Figure 10:**
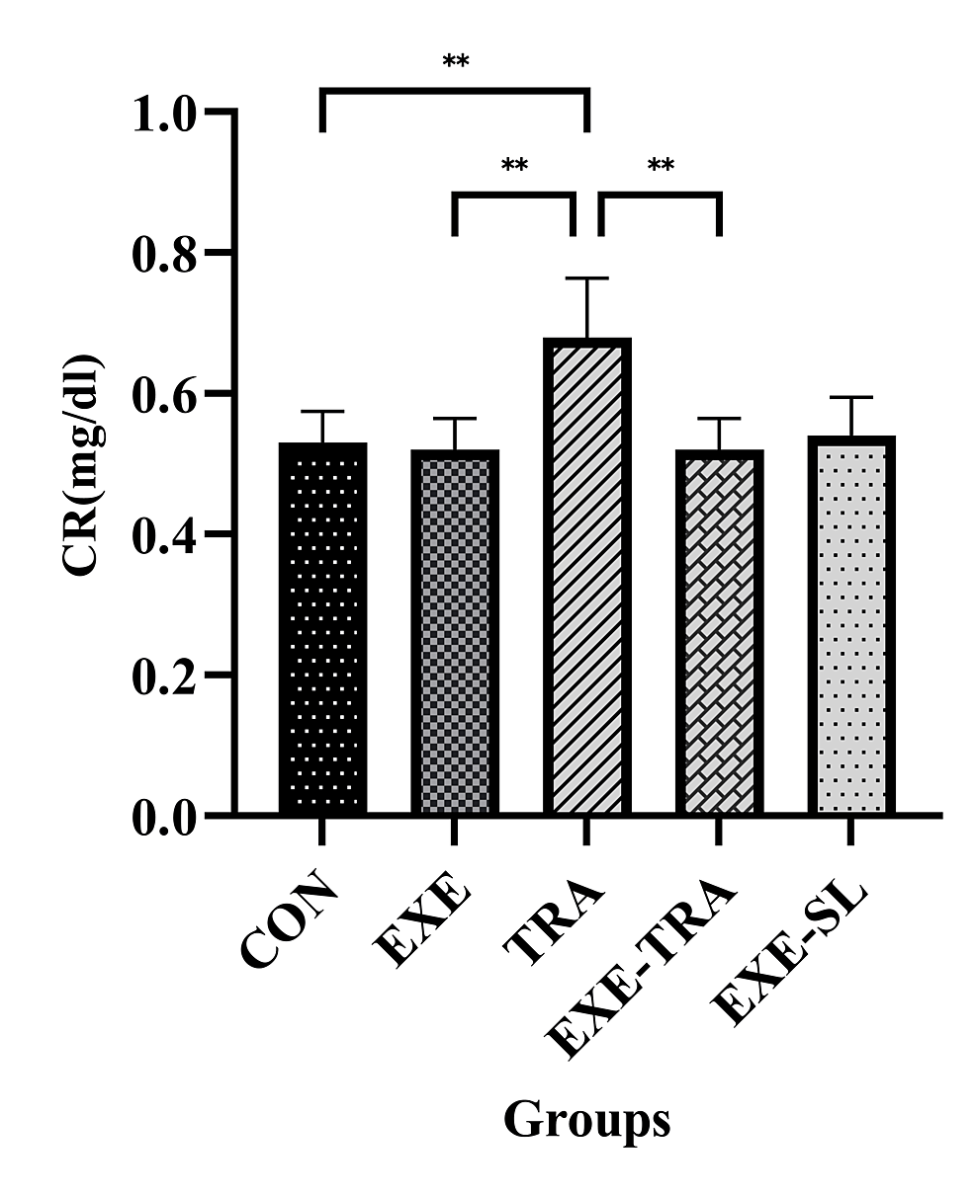
Effect of eight weeks of HIIT on CR in serum Data are presented as mean ± SD for n=5 in each group. Our findings demonstrate a notable rise in CR levels within the TRA group in comparison to all other groups. Additionally, EXE contributed to a reduction in urea levels within the EXE-TRA group when compared to the TRA group, with no discernible difference noted between the EXE-TRA and CON groups. ** p<0.01, CON: control, EXE: exercise, TRA: tramadol, EXE-TRA: exercise-tramadol, EXE-SL: exercise-normal saline

Our results showed that urea and CR levels in the TRA group significantly increased compared to the CON (p<0.0001 and p<0.01) and EXE groups (p<0.0001 and p<0.01). Although a significant decrease in urea and CR levels was observed in the EXE-TRA group compared to the TRA group (p<0.0001 and p<0.01), no difference was observed between the EXE-TRA and CON groups. This suggests that HIIT can decrease CR and urea levels after TRA administration to the levels of the CON group (Figures 9-10).

Effect of HIIT on pathological changes of kidney tissue following eight weeks of TRA administration

**Figure 11:**
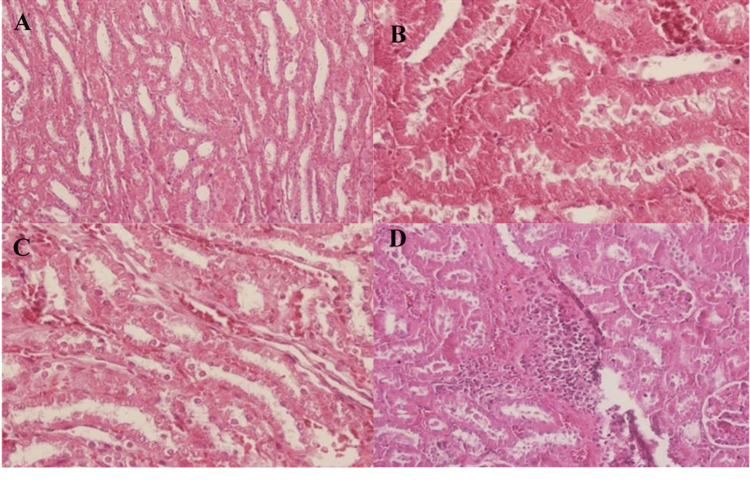
Histopathological view of the renal section in H&E A: CON (×200), B: TRA (×400), C: EXE (×400), and D: EXE-TRA (×200) groups as described in the Materials and Methods section. The CON group showed normal kidney structures (A). The TRA group exhibited severe necrosis, moderate hyaline casts, mild hyperemia, and minimal interstitial nephritis (B). The EXE group had no necrosis or nephritis but showed severe hyperemia and mild inflammatory cell infiltration (C). In the EXE-TRA group, EXE reduced the severity of hyaline casts and necrosis compared to the TRA group (D). However, hyperemia and interstitial nephritis increased, and inflammatory cell infiltration was observed in 25-75% of the EXE-TRA group. H&E: hematoxylin and eosin, CON: control, EXE: exercise, TRA: tramadol, EXE-TRA: exercise-tramadol, EXE-SL: exercise-normal saline

The microscopic findings of the CON group showed normal glomeruli, cortical and medullary tubules, interstitial spaces, and vessels (Table 2 and Figure 11A).

**Table 2:** Degrees of damage in kidney tissue of different groups Histopathology of kidney tissue was examined in a bright field microscope in groups of rats as prescribed in the Materials and Methods section. The severity of damage was scored as follows: 0 - no damage, 1 - minimal (0-5% involvement), 2 - mild (5-25% involvement), 3 - moderate (25-75% involvement), and 4 - severe (75-100% involvement). CON: control, EXE: exercise, TRA: tramadol, EXE-TRA: exercise-tramadol, EXE-SL: exercise-normal saline

	CON	EXE	TRA	EXE-TRA	EXE-SL
Glomerular atrophy	0	0	0	0	0
Hyaline casts in the tubular lumen	0	1	3	2	1
Interstitial nephritis	0	0	1	2	0
Hyperemia	0	4	2	3	4
Infiltration of inflammatory cells	0	2	0	3	1
Apoptosis or necrosis	0	0	4	3	0

In the TRA group, severe necrosis without inflammation was evident. The presence of hyaline casts was moderate, hyperemia was mild, and minimal interstitial nephritis was observed (Figure 11B). In the EXE group, necrosis and nephritis were absent, while severe hyperemia and mild infiltration of inflammatory cells were observed (Table 2 and Figure 11C).

It appears that EXE decreased the severity of hyaline casts from moderate (in the TRA group) to a mild level in the EXE-TRA group and reduced the severity of necrosis from severe to moderate. However, hyperemia and interstitial nephritis were increased in the EXE-TRA group compared to the TRA group (Table 2). Nevertheless, infiltration of inflammatory cells (an indicator of inflammation) ranging from 25% to 75% was evident in the EXE-TRA group (Table 2 and Figure 11D).

Inflammation with pigment deposition and hemorrhage was present in all exercise groups (EXE, EXE-TRA, and EXE-SL). However, degeneration and apoptosis of the tubular cells did not occur in the EXE groups, as observed in the groups that received TRA (TRA and EXE-TRA groups), as shown in Table 2.

## Discussion

TRA is an opioid analgesic widely prescribed for moderate-to-severe pain; however, previous studies have reported adverse effects, including kidney damage, associated with chronic TRA use or overdose. The present study showed a significant increase in urea and CR levels, as well as a decrease in Alb levels, in all animals treated with chronic use of TRA. Conversely, MDA as an oxidative stress marker was increased, while antioxidant indices such as SOD, TAC, and GPx were decreased following repetitive doses of TRA. However, our data showed that HIIT training reversed these kidney function indicators, as well as oxidative stress and antioxidant markers, to normal levels, thereby improving kidney function.

In Figure 4, our data showed that TRA increased MDA levels. This increase in MDA generation may be attributed to a significant decrease in mitochondrial electron transport chain complexes I, III, and IV, which might explain the increased MDA generation via TRA consumption, as previous studies have suggested [20,21]. ROS and oxidative stress have been implicated in various signaling pathways, including cell growth, mitogenesis, degradation, inflammation, and apoptosis. Oxidative stress is also considered an inducer of cellular damage, which is mainly counteracted by antioxidant defense systems [11]. Consistent with this, our results showed that TRA administration significantly reduced GPX and SOD levels while increasing MDA levels in kidneys, as shown in a previous study [6]. Excessive oxidative stress can also activate various signaling pathways, such as mitogen-activated protein kinase (MAPK), NF-κB, and NLRP3. It is known that MAPK signaling pathways, including ERK 1/2, JNK 1/2/3, and p38, are involved in cell proliferation, differentiation, apoptosis, and degeneration [20,21]. Similar findings were observed in the pathology report of this study (Figure 11 and Table 2).

On the other hand, HIIT training, whether performed individually or in combination with TRA, induced a significant difference in MDA, SOD, TAC, and GPX compared to the TRA group. It is known that EXE intensity shifts redox balance toward oxidative stress, which is necessary for the initial adaptation that supports EXE-induced hormesis. HIIT increases antioxidant capacity compared to low and moderate EXE intensities without causing changes in lipid peroxidation [22,23]. In agreement with our results, other studies indicated that HIIT significantly enhanced the effectiveness of antioxidant defense mechanisms and reinforced the body's capacity to eliminate pro-oxidants [23], demonstrating that regular HIIT can decrease pro-oxidants such as MDA [24]. The HIIT training increased GPX and SOD in those who received TRA (EXE-TRA) compared to the TRA group and reversed the destructive effect of TRA on the kidneys. The mechanism of the protective effect of HIIT is probably due to the increased expression of PGC1-α, which activates enzymes such as GPX and MnSOD by transcribing combining-protein factors of CREB [14,25,26]. The molecular mechanism behind this activation might be the combination of PGC1-α with ERR α and the activation of SIRT3 in the mitochondria matrix. SIRT3 can regulate ROS production by binding and deacetylating mitochondria complexes I and II, leading to increased antioxidant activity by deacetylating MnSOD. Although PGC1-α is considered a potent suppressor of ROS production, overexpression of PGC1-α can increase antioxidant defense by upregulating MnSOD expression and catalase enzyme activity [27].

In contrast to our study, a previous study has shown that pretreatment with TRA decreased dose-dependently serum levels of IL-6 after incisional operation in rats [28]. Additionally, another study has shown that IL-6 as a proinflammatory cytokine is produced immediately after injury; thus, a single dose of TRA was able to decrease its levels [28], but in our study, animals received repetitive doses of TRA for a long period of two months. After repetitive administration, it was demonstrated that the immune modulatory effect of TRA disappeared, and it is no longer considered as immunosuppressive as other opioids [29]. Our study has shown that long-term TRA administration to rats did not change even IL-10. Also, in a study on dogs with innate immune system function, TRA did not affect IL-10, although its main metabolite, O-desmethyl TRA, was able to decrease IL-10 levels [30]. Considering eight weeks of continuous TRA administration in our study, the apparent decrease of IL-10 and IL-6 was probably due to TRA metabolites and not TRA itself. This suggests that chronic use of TRA might have a positive impact on the immune system, probably through suppressing the production of pro-inflammatory cytokines [28,29,31]. Furthermore, our findings have indicated that EXE significantly increased both IL-6 and IL-10 compared to the TRA group. The increase in IL-10 induced by EXE could potentially prevent chronic low-grade inflammation and tissue damage. In fact, temporary hypoxia caused by HIIT training has been found to be a strong stimulus for increasing IL-6 levels [32,33]. Moreover, EXE itself was able to restore the decreased level of IL-6 produced by TRA. The duration, intensity, and recovery time of EXE are other factors that determine the kinetics of IL-6. Even 48 hours after EXE training, IL-6 levels remain higher than resting levels, which can cause reduced glycogen availability and changes in calcium homeostasis due to MAPK stimulation [33]. In contrast, in CKD patients, EXE was considered an anti-inflammatory therapy [15].

In agreement with our results, previous studies have shown that TRA significantly increased serum levels of CR and urea while decreasing serum levels of Alb [6], indicating its potential renal damage and impaired kidney function. The elevation in blood urea levels could also be caused by the destruction of red blood cells or the presence of toxic compounds [6,11,34]. The moderate presence of hyaline casts and severe necrosis found in the histopathology of kidney tissue, consistent with these biochemical findings, can explain why TRA negatively affected nephron function and caused kidney damage. Controversially, HIIT reversed the effect of TRA and decreased the serum levels of urea and CR to CON. Although the precise mechanisms by which EXE affects renal indicators are not fully understood, researchers have demonstrated that increasing subjects' aerobic capacity reduces their reliance on phosphoryl compounds with high energy-producing capabilities during physical activity [35]. This adaptation is associated with decreased adenosine monophosphate deaminase activity and reduced uric acid production [36]. Additionally, EXE can enhance kidney function by increasing nitric oxide availability, reducing oxidative stress, and elevating antioxidant levels in kidney tissue [14,36]. As a result, regular and continuous EXE adaptations can normalize blood urea, CR, and Alb levels. Lower blood urea levels indicate increased glomerular filtration and urea excretion through urine or decreased renal tubular reabsorption [37,38]. The urea transport rate is determined by the electrochemical gradient of diffusion across the membrane and its permeability to the material [38]. Additionally, glomerular filtration depends on the negative charge of the podocyte basement membrane, which contains negatively charged molecules. Therefore, changes in the electrical potential of tubular and glomerular cell membranes due to HIIT EXEs may affect renal filtration and urea absorption [38,39].

Furthermore, our findings are supported by the histopathological results. HIIT significantly increased inflammatory cytokines. Indeed, inflammatory cell infiltration, pigment deposition, and hemorrhage were seen in all EXE groups (Table 2). Although TRA did not affect IL-6 and IL-10 compared to the CON group, when combined with EXE (EXE-TRA), moderate inflammation and hyperemia were assessed in kidney tissue. This was the result of increased inflammation induced by HIIT, which leads to hyperemia. It is known that inflammation increases blood flow into tissues through the dilation of arterioles [40].

Our biochemical findings demonstrated that TRA increased urea and CR levels, while EXE decreased them. Pathological analysis also revealed that EXE reduced the moderate formation of hyaline casts in the tubular lumen, indicating potential benefits to kidney health (TRA and EXE-TRA groups in Table 2). Hyaline casts are composed of urinary particles wrapped in a protein matrix, and their presence can indicate kidney function [34,41]. It appears that TRA caused minimal interstitial nephritis, which was worsened slightly by EXE in the EXE-TRA group (Table 2). It is partially due to hyperemia and pigment deposition caused by EXE [42]. In agreement with other studies and our pathological finding, TRA increases apoptosis [22], while EXE can improve the apoptosis rate [42,43]. EXE training is recommended as an effective way to slow down the process of apoptosis by activating the 1-Mcl-cGMP-NO-iNOS pathway [42]. Also, a previous study has shown that physical activity stimulated multiple pathways and activated the protein PGC1α, resulting in changes in mitochondrial status [43]. As a result, this leads to a decrease in the production of ROS and an increase in mitochondrial resistance to permeability and apoptotic signaling [13].

Limitations

While this study offers valuable insights into the protective effects of HIIT, it is important to acknowledge its limitations. Firstly, the use of an animal model restricts the ability to fully replicate the intricate physiological complexity and pathology of the human kidney. Moreover, the controlled environments utilized in this study may not accurately reflect the complexities of real-world conditions. Secondly, considering the heightened inflammatory factors observed following intense EXE, it would have been beneficial to explore the effects of moderate-intensity interval training as well. Thirdly, the underlying mechanisms of the observed effects were not fully examined, warranting further molecular research. Additionally, this study did not investigate the role of pre-treatment EXE (a period of EXE prior to TRA administration).

## Conclusions

Treating rats with TRA for a prolonged period of two months resulted in increased levels of oxidative stress, urea, and CR, as well as decreased levels of antioxidants and Alb. The presence of severe apoptosis and moderate hyaline casts in kidney tissue confirmed these findings. However, no significant changes in inflammatory cytokines were observed. In contrast, concomitant HIIT with TRA improved biochemical indicators and reduced oxidative stress markers, thereby reversing tissue impairment, including apoptosis. It is worth mentioning that HIIT, regardless of the presence of TRA, increased inflammatory cytokines and exacerbated inflammatory cell infiltration in the kidney in all EXE groups. Probably, long and continuous use of TRA or its metabolites might alleviate HIIT-induced inflammation. Finally, it was concluded that chronic usage of TRA could potentially lead to kidney damage, which is alleviated by HIIT.
